# The Integral Role of Physiotherapy in Combined Complete Anterior Cruciate Ligament and Posterior Cruciate Ligament Arthroscopic Reconstruction: A Case Report

**DOI:** 10.7759/cureus.57556

**Published:** 2024-04-03

**Authors:** Vaishnavi R Waghe, Priya Tikhile, Deepali S Patil

**Affiliations:** 1 Musculoskeletal Physiotherapy, Ravi Nair Physiotherapy College, Datta Meghe Institute of Higher Education and Research, Wardha, IND

**Keywords:** rehabilitation, physiotherapy, posterior cruciate ligament reconstruction (pclr), anterior cruciate ligament repair (aclr), posterior cruciate ligament (pcl), anterior cruciate ligament (acl)

## Abstract

Combined anterior cruciate ligament (ACL) and posterior cruciate ligament (PCL) injuries are infrequent in clinical practice, often leading to severe knee instability and functional limitations. A 30-year-old male presented with right knee pain and swelling following a two-wheeler accident. Diagnostic investigations confirmed complete ACL and PCL tears. The surgical intervention comprised arthroscopic-assisted ACL reconstruction using semitendinosus and gracilis tendons, accompanied by arthroscopic PCL reconstruction. Postoperatively, structured physiotherapy rehabilitation was initiated. After 12 weeks of rehabilitation, significant improvements in range of motion and muscular strength were observed. Tailored physiotherapy facilitated prompt recovery, enhancing functional mobility and independent ambulation. This case highlights the efficacy of comprehensive surgical intervention followed by structured rehabilitation in achieving favorable outcomes in patients with combined ACL and PCL injuries. Tailored physiotherapy plays a crucial role in optimizing functional recovery and facilitates the enhancement of the patient's functional mobility and independent ambulation.

## Introduction

Multiple ligamentous injuries involving the anterior cruciate ligament (ACL) and posterior cruciate ligament (PCL) are rare occurrences but entail excruciating pain. Patients with concurrent ligament injuries commonly report incapacitating knee instability, severely limiting their daily activities [1]. Excessive tension overload in the PCL and abrupt posterior translation to the flexed knee are the primary mechanisms leading to posterior cruciate ligament injuries [2]. The ACL and PCL function synergistically as the primary barriers against both anterior and posterior tibial translation, and they significantly contribute to the knee's rotatory stability, facilitating varus and valgus tibial rotation [3]. PCL injuries frequently coincide with concomitant ACL and PCL injuries, characterized as bi-cruciate injuries or Schenck-type Knee Dislocation II [4]. The ACL originates at the medial aspect of the lateral femoral condyle and terminates near the anterior horn of the lateral meniscus at the middle of the tibia plateau. The ACL's anatomical structure comprises two functional bundles: the posterolateral (PL) and anteromedial (AM) bundles. The ACL's flat, oval shape is crucial for adapting to the knee's changing flexion axes and stabilizing the knee joint at various flexion angles. Studies have demonstrated that the PL bundle stabilizes anteroposterior and rotational pressures at near-to-extension postures of less than 30°, while the AM bundle becomes tensioned and functional at higher flexion angles [5].

The principal function of the PCL is to resist posterior tibial translation with respect to the femur. Comprising two separate bundles, the PCL operates in a codominant relationship, providing both rotatory stability and supplementary support. The anterolateral (AL) and posteromedial (PM) bundles exhibit distinct spatial orientations, with the AL bundle having a higher vertical orientation. The PM bundle serves as the primary constraint for posterior tibial translation during intermediate flexion, while the AL bundle predominates during extension and deep flexion [6]. Understanding the healing and rehabilitation processes following anterior cruciate ligament repair (ACLR) and posterior cruciate ligament reconstruction (PCLR) is paramount for restoring knee function [7]. Research indicates that the quadriceps muscle of the injured leg exhibits greater strength and resilience following a ruptured PCL compared to an ACL rupture [8]. Conversely, in ACLR legs with hamstring autograft, the hamstring remains relatively robust compared to the uninvolved leg, while the hamstring of PCLR legs with allograft significantly weakens compared to the unoperated limb after two years [9].

ACL and PCL reconstruction done simultaneously is technically challenging, but it is attainable and produces good to exceptional functional outcomes [7]. Conservative treatment can effectively treat knees that have an initial PCL injury along with concurrent articular deterioration [8]. Surgery is necessary for combined harm to the posterolateral corner and the ACL or PCL. The best chance of obtaining satisfactory functional results is with combined reconstruction [10]. Following reconstruction, the patient's range of motion is momentarily restricted, and the flexor and extensor muscles in the knee joint lose strength. It is expected that both in static and dynamic settings, the muscle strength values should recover to preoperative levels upon completion of the full physiotherapy program. Restoring the muscle groups that make up the knee joint's antagonist strength ratio is one of the objectives of physical therapy following ACL surgery. The question of when and how much muscle strength may be safely regained must be considered when designing physiotherapy treatments [11]. Following ACL reconstruction, patients can choose from a number of well-liked rehabilitation programs. In addition to relying on shared presumptions, these programs vary in the exercises they offer and in the amount and timing of the load applied [12].

## Case presentation

Patient information

**Figure 1:**
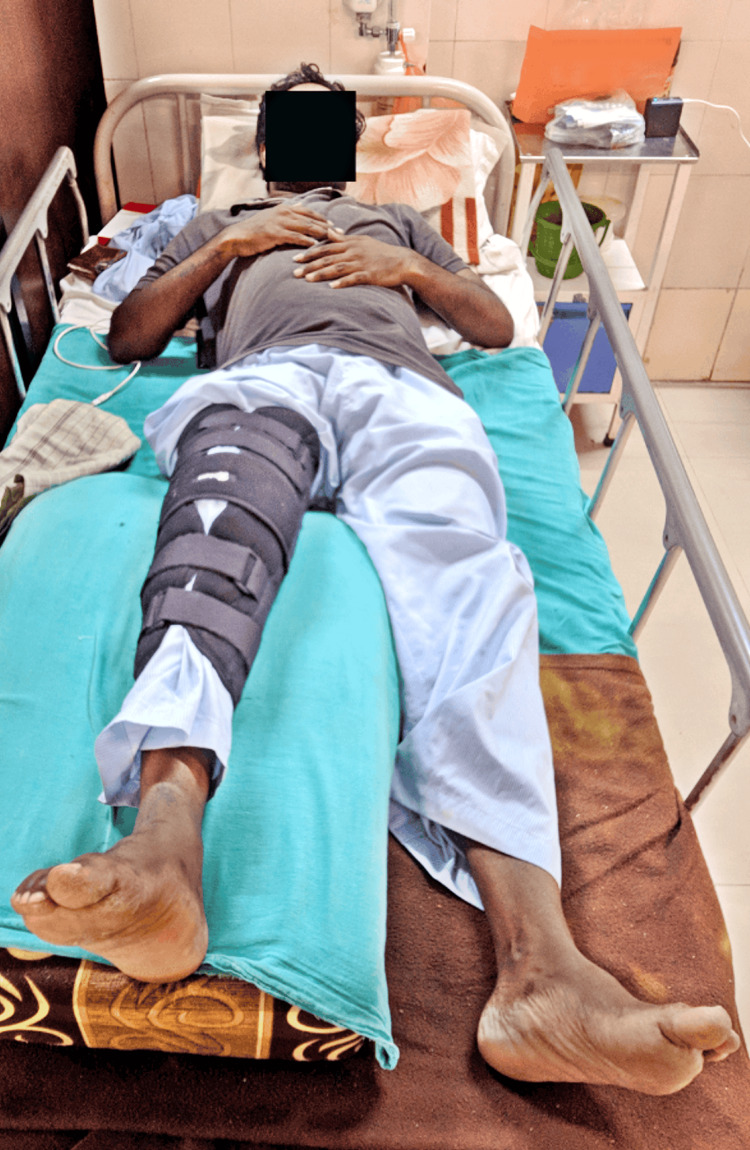
Patient in a supine lying position with the right lower limb elevated

A 30-year-old male patient presented one month ago with complaints of a twisting injury to his right knee sustained during a fall from a two-wheeler. Following the incident, the patient was initially able to ambulate without difficulty. However, he experienced the onset of pain and swelling, which was manifested diffusely across the entirety of the right knee, exacerbating with movements and alleviating with rest, elevation, and prescribed medications. In response to these symptoms, the patient sought medical attention at a private hospital where investigations were done and medicines were given. Then, the patient subsequently presented to our hospital for further evaluation and management where an X-ray was performed. The patient received recommendations for oral medications and underwent magnetic resonance imaging (MRI), which showed a complete tear of the ACL and PCL of the right side, for which arthroscopic-assisted ACLR using semitendinosus and gracilis tendon on the right side was done along with soft tissue reconstruction by arthroscopic PCLR right side. Upon physiotherapy assessment and investigations, the patient was seen in a supine lying position as shown in Figure 1. The visual analogue scale (VAS) revealed a pain score of 8 out of 10 during flexion movement and 3.8 out of 10 at rest. A sudden onset, severe intensity, and sharp shooting quality characterized the pain. Notably, the pain exhibited a non-progressive nature and lacked diurnal variation. Furthermore, it was exacerbated by movement and alleviated with rest and the administration of prescribed medications.

Clinical findings

**Figure 2:**
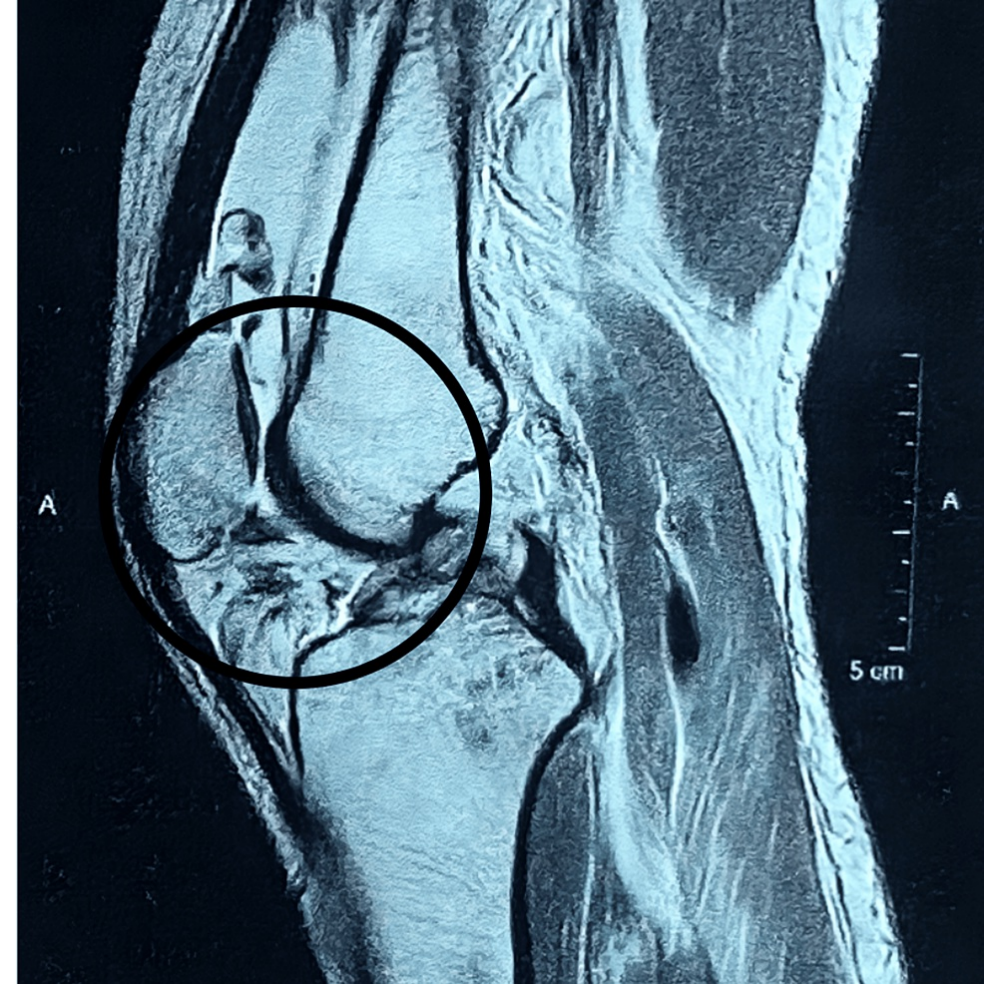
MRI showing ACL tear MRI: Magnetic resonance imaging; ACL: Anterior cruciate ligament

**Figure 3:**
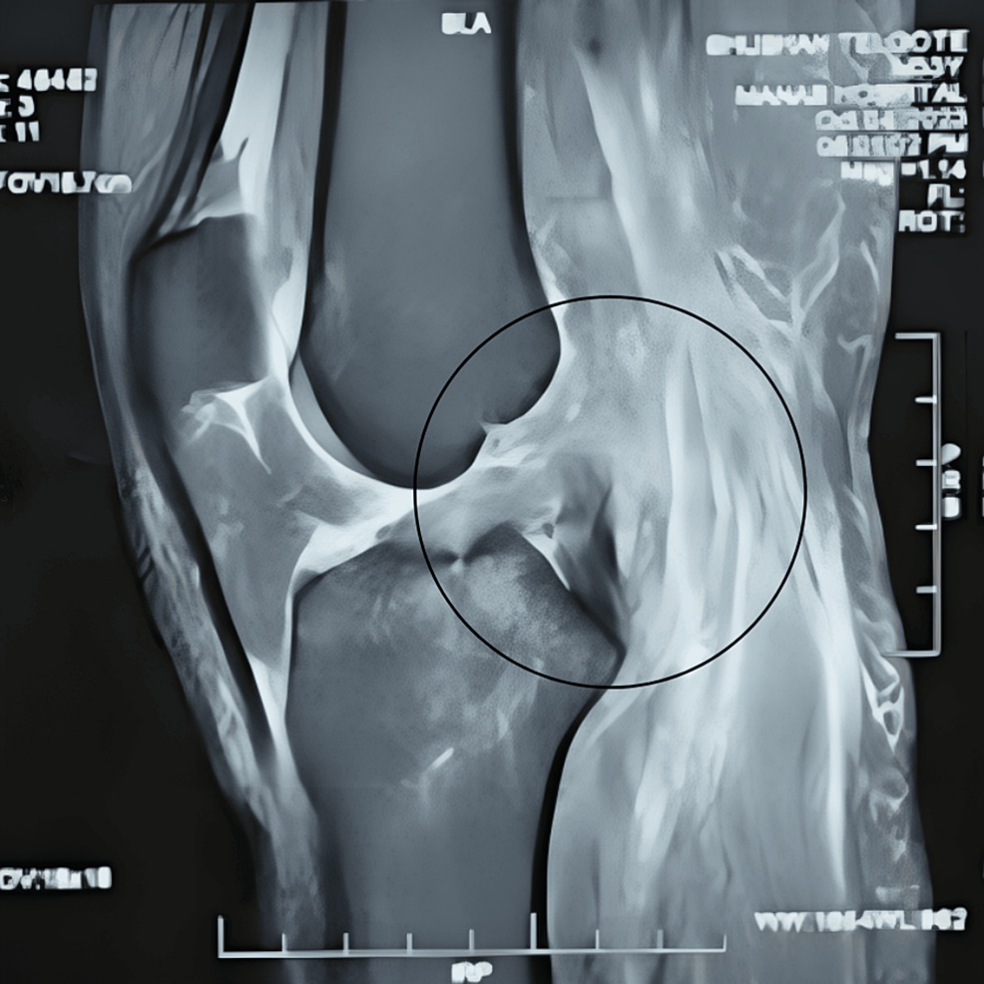
MRI showing PCL tear MRI: Magnetic resonance imaging; PCL: Posterior cruciate ligament

**Figure 4:**
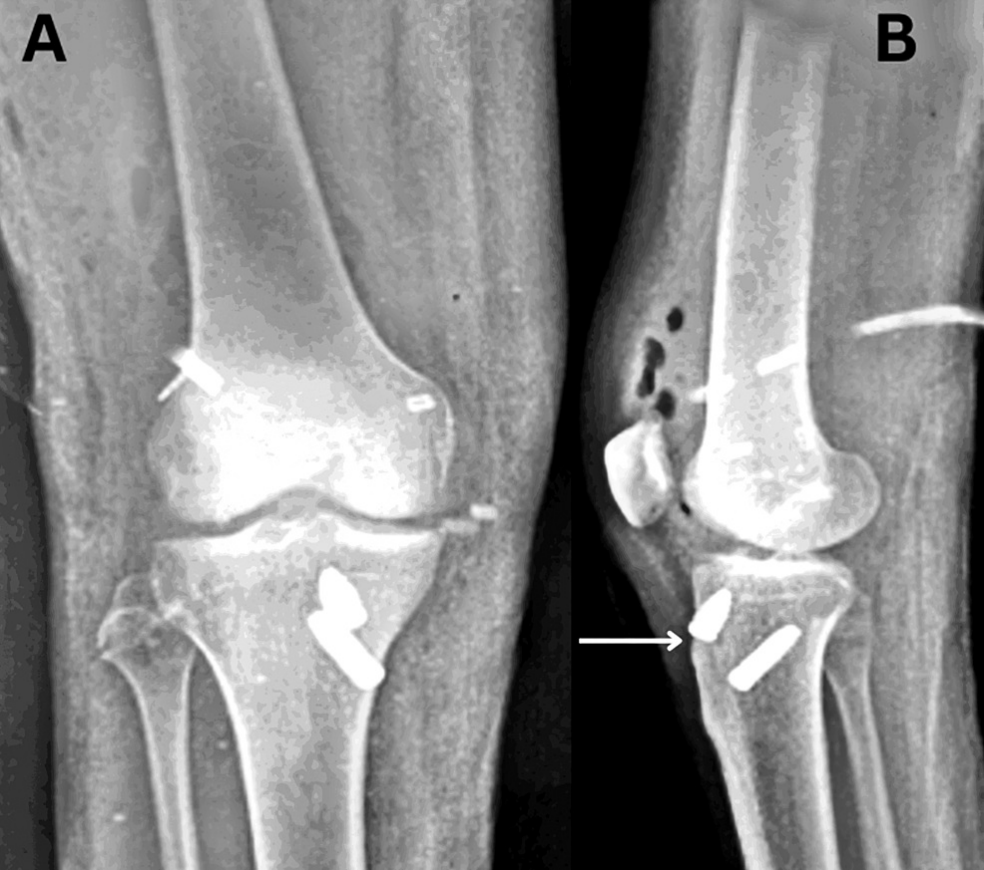
X-ray (A) Posteroanterior view; (B) Lateral view showing arthroscopic reconstruction

Following the acquisition of informed consent, a comprehensive examination of the patient was conducted. According to the information provided by the patient, he reported a twisting injury to his right knee sustained during a fall from a two-wheeler occurring over the past month. The patient underwent examination in a supine position, with both anterior superior iliac spines (ASIS) aligned at an equal level. The underlying skin exhibited normal characteristics with an absence of swelling. Notably, a deformity was observed, accompanied by muscle wasting in the right thigh, and limb length discrepancy was identified. Vital signs were within normal limits. Local examination revealed Grade II tenderness in the right lower limb at the knee. Manual muscle testing (MMT) was employed during the physical examination, revealing noteworthy findings. Strength assessment in the right lower extremities demonstrated a rating of one out of five for both knee flexion and extension. Moreover, a decrease in the range of motion was also noted. MRI findings are illustrated in Figures 2-3, and postoperative X-ray images in Figure 4.

Therapeutic intervention

Physiotherapy rehabilitation protocol was given in three phases for 12 weeks as shown in Table 1.

**Table 1:** Therapeutic interventions for 12 weeks Reps: Repetitions; CPM: Continuous passive motion; PWB: Partial weight bearing; FWB: Full weight bearing

GOALS	PHASE I (0-4 weeks)	PHASE II (4-8 weeks)	PHASE III (8-12 weeks)
Reduce pain, restoration of movement as normal as possible, increase strength of the affected muscles, increase endurance, restoration of kinesthesia/proprioception mechanism, restoration of functional activities	Cryotherapy	Transcutaneous electrical nerve stimulation	Active assisted to active resisted exercises
	Limb elevation	Pressure bandage	Ambulation with walker from partial to FWB
	Isometrics: static quadriceps, static hamstrings, static glutes (10 reps, 2 sets)	Transverse friction massage	Dynamic quads, semi squats, half lunges for kinesthesia/proprioception restoration
	Cast for immobilization	Active exercises for affected joints began slowly and progressed gradually	
	Upper limb strengthening exercises	Isometrics vigorously	
	Ambulation with walker non-weight bearing for 3-4 weeks	Bilateral heel slides	
		CPM for 10 minutes	
		Passive range of motion exercises	
		Ambulation with walker from non-weight bearing to PWB	

Progression would be done to improve the strength, balance, and flexibility with functional activities by increasing the sets, repetitions, and resistance or by decreasing the rest time.

Follow-up and outcome measures

The follow-up evaluation of manual muscle testing at the 12-week is presented in Table 2, while the range of motion (ROM) data is shown in Table 3. Comprehensive outcome measures, both pre-treatment and post-treatment, are detailed in Table 4.

**Table 2:** Manual muscle testing of the right lower extremity pre and post-rehabilitation 3: Complete ROM against gravity; 3+: Complete ROM against gravity with slight resistance; 4: Complete ROM against gravity with moderate resistance; 4+: Complete ROM against gravity with nearly full resistance ROM: Range of motion

Variables	Measurement	Before treatment	After treatment
Manual muscle testing - Hip	Flexors	3+	4
	Extensors	3	4
	Adductors	3+	4
	Abductors	3	3+
Knee	Flexors	3	3+
	Extensors	3+	4
Ankle	Planter flexors	3+	4
	Dorsiflexors	3+	4+

**Table 3:** ROM of the right lower extremity pre and post-rehabilitation ROM: Range of motion

Variables	Measurement	Before treatment	After treatment
Hip	Flexion	0-45	0-85
	Extension	0-10	0-15
Knee	Flexion	0-45	0-90
	Extension	45-0	90-0
Ankle	Dorsiflexion	0-20	0-25
	Plantar flexion	0-10	0-20

**Table 4:** Outcome measures pre and post-rehabilitation Visual analogue scale: 8 - severe, 4 - moderate; Lower extremity function scale: 35 - moderate to severe functional limitation, 70 - normal or minimal functional limitation; IKDC-SKF: 0-100, higher scores indicate higher levels of function and fewer symptoms; European-5D QOL questionnaire: 1 - worst imaginable health status, 15 - best imaginable health status IKDC-SKF: International Knee Documentation Committee subjective knee form; European (5D) QOL: European five-dimension quality of life

Outcome measures	Pre-treatment	Post-treatment
Visual analogue scale	8/10	4/10
Lower extremity function scale	35/80	70/80
IKDC-SKF	26/100	79/100
European (5D) QOL questionnaire	5/15	13/15

## Discussion

Patients undergoing ACL reconstruction and those receiving graft-donor knees derive significant advantages from tailored rehabilitation programs. The specific principles of such programs aim to facilitate the attainment of knee symmetry. These principles include eliminating time frames as postoperative guidelines, enabling immediate unrestricted ROM, advocating for bed rest during the initial postoperative week, and implementing additional measures. With regard to minimizing postoperative problems after ACL reconstruction, the knee symmetry model yields results that maximize patients' short and long-term outcomes [13]. According to Lee et al., one year following ACLR with an autologous hamstring tendon, the knee flexor strength recovered to 80% of the unaffected leg's strength [14]. According to Keays et al., following ACLR with a hamstring autograft, hamstring muscular strength recovered more slowly than quadriceps muscle strength at a six-month follow-up [15]. Regardless of the kind of graft utilized, patients following ACLR have been advised to undergo expedited rehabilitation, which includes isokinetic flexor strengthening, in order to prevent muscle weakness [16].

A recent comprehensive analysis of randomized controlled trials with defective or rebuilt ACLs indicated that extensive rehabilitation is necessary to get a good surgical outcome. At least six weeks following the treatment or injury is the ideal time to start open kinetic chain activities [17]. Traditionally, PCLR has been treated with a more cautious rehabilitation [18]. In order to strengthen muscles following PCLR, several rehabilitation strategies have been proposed recently [19]. Closed-chain kinetic workouts might begin as soon as possible after surgery or up to 12 weeks later [20]. For the purpose of strengthening the flexors, active hamstring activities should be postponed for at least 12 weeks following PCLR, although quadriceps workouts are recommended because of the agonistic nature of the PCL [21].

## Conclusions

The present case study underscores the significance of tailored physical treatment regimens after combined ACL and PCL reconstructive surgeries to optimize healing and restore knee stability. Following a series of training regimens, knee discomfort was reduced, muscular strength improved, and active range of motion (AROM) increased. Movements such as closed-chain movements, knee bending, co-contractions, and AROM produced noteworthy effects, concluding that a well-thought-out physical treatment program along with appropriate ergonomic guidance and medication can decrease discomfort and improve strength and ROM in the muscles with an improved general quality of life of the patient.
